# Osteopathic Manipulative Treatment for Pediatric Conditions: An Update of Systematic Review and Meta-Analysis

**DOI:** 10.3390/jcm11154455

**Published:** 2022-07-30

**Authors:** Pawel Posadzki, Bhone Myint Kyaw, Arkadiusz Dziedzic, Edzard Ernst

**Affiliations:** 1 Kleijnen Systematic Reviews, 6 Escrick Business Park, York YO19 6FD, UK; 2 Lee Kong Chian School of Medicine, Nanyang Technological University, 50 Nanyang Ave, Singapore 639798, Singapore; bhonemkyaw@gmail.com; 3 Department of Restorative Dentistry with Endodontics, Medical University of Silesia, 40-0055 Katowice, Poland; adziedzic@sum.edu.pl; 4 Complementary Medicine, University of Exeter, 25 Victoria Park Road, Exeter EX2 4NT, UK; e.ernst@exeter.ac.uk

**Keywords:** osteopathy, pediatric conditions, systematic review, meta-analysis

## Abstract

Osteopathic manipulative treatment (OMT) continues to be used for a range of diseases in children. Objectives: The aim of this paper is to update our previous systematic review (SR) initially published in 2013 by critically evaluating the evidence for or against this treatment. Methods: Eleven databases were searched (January 2012 to November 2021). Study selection and data extraction: Only randomized clinical trials (RCTs) of OMT in pediatric patients compared with any type of controls were considered. The Cochrane risk-of-bias tool was used. In addition, the quality of the evidence was rated using Grading of Recommendations, Assessment, Development and Evaluation (GRADE) criteria, as recommended by the Cochrane Collaboration. Results: Thirteen trials met the eligibility criteria, of which four could be subjected to a meta-analysis. The findings show that, in preterm infants, OMT has little or no effect on reducing the length of hospital stay (standardized mean difference (SMD) −0.03; 95% confidence interval (CI) −0.44 to 0.39; very low certainty of evidence) when compared with usual care alone. Only one study (8.3%) was judged to have a low risk of bias and showed no effects of OMT on improving exclusive breastfeeding at 1 month. The methodological quality of RCTs published since 2013 has improved. However, adverse effects remain poorly reported. Conclusions: The quality of the primary trials of OMT has improved during recent years. However, the quality of the totality of the evidence remains low or very low. Therefore, the effectiveness of OMT for selected pediatric populations remains unproven.

## 1. Introduction

In 2021, there were almost 135,000 doctors of osteopathy (DOs) in the US, amounting to approximately 11% of the physician workforce in the US [1]. During their training, DOs spend 300–500 h learning the principles of osteopathic manipulative treatment (OMT), which constitutes a wide range of manual techniques aimed at “supporting homeostasis and improving physiologic function of interconnected system of nerves, muscles and bones” [2]. The use of OMT among American DOs continues to decline [3,4]. However, outside the US, OMT remains the hallmark therapy of osteopaths, and OMT is often recommended for the treatment of pediatric conditions [5].

The effectiveness of OMT for pediatric conditions is questionable. Our systematic review (SR) in 2013 concluded that the evidence was insufficient to prove its effectiveness [6]. Since then, numerous studies have been published on the topic with conflicting results [5,7,8,9]. A review by Parnell Prevost et al. (2019) suggested that inconclusive but favorable evidence does exist [7]. Subsequently, Yu et al. 2021 [10] identified several methodological flaws in the conduct of this review, and a recent scoping review concluded that “no strong clinical recommendations can be made; and high-quality, scientifically rigorous research is required to evaluate safety, feasibility, and efficacy in pediatric populations” [8].

The rationale for this update SR is at least twofold. First and foremost, more than a dozen randomized clinical trials (RCTs) have been published since 2013. Secondly, the incorporation of the GRADE method has improved the clarity of the findings [11]. Therefore, the aim of this SR is to update our previous SR (2013) by focusing on OMT rather than treatments delivered by DOs. 

## 2. Materials and Methods

The methodology of the update followed that of the originally published paper [6], the recommendations of the Cochrane Collaboration [12], the Centre for Reviews and Dissemination [13], and the Preferred Reporting Items for Systematic Reviews and Meta-analyses (PRISMA) guidelines [14] (Supplementary Materials FileS1 ). All RCTs (published and unpublished) evaluating the effectiveness of OMT in pediatric conditions, i.e., children and adolescents aged 18 or below, were eligible. We considered all types of control groups. There were no restrictions in eligibility criteria based on study characteristics or sources of information, i.e., time or language. 

### 2.1. Data Source and Search Strategy

Eleven databases were searched (from November 2012 to November 2021): AMED, CINAHL, Embase, MEDLINE, OSTMED.DR, PsycINFO, The Cochrane Library, ISI Web of Knowledge, Osteopathic Research Web, PEDro, and Rehabdata using the same search strategy (also translated to other databases) (Supplementary Materials FileS2 ). The reference lists of the located articles and key SRs of OMT were manually searched for further relevant studies. Relevant characteristics related to pediatric patients, OMT, comparators, outcome measures, and results (effectiveness of the interventions) were extracted by the first author (PP) and validated by two other authors (BMK and AD) using a custom-made data extraction form. Any disagreements were settled through a discussion. 

### 2.2. Risk of Bias Assessment 

We assessed the methodological risk of bias of the included studies using the Cochrane Collaboration’s risk-of-bias tool [10]. The overall risk of bias was assessed by the first author (PP) and validated by the other authors (BMK and AD), with disagreements settled through discussions.

### 2.3. Data Synthesis and Assessment of Heterogeneity Sensitivity Analysis

Where studies were similar in terms of populations, interventions, comparators, outcome measures, and primary endpoints, statistical pooling of the data using a random-effect model was performed. Where substantial clinical or methodological heterogeneity was detected, we did not pool results but instead used a narrative data synthesis. The degree of heterogeneity was visually inspected by looking at forest plots; examining the Chi2 test for heterogeneity; and calculating the I2 statistic. An I2 value of ≥50% represents a substantial amount of heterogeneity. When substantial heterogeneity was detected in pooled results, to further explore the reasons for variability, we conducted sensitivity analyses by removing studies with a high risk of bias. 

### 2.4. Summary of Findings 

Summary of findings tables were prepared to present the results for the main outcomes based on pooled studies. The overall quality of the evidence was assessed by the first author (PP) and validated by two other authors (BMK and AD) using the GRADEprofiler (GRADEpro 2016) software. To evaluate the overall quality of the evidence, the following criteria were considered (and downgraded where appropriate): limitations of studies (risk of bias), inconsistency of results, indirectness of the evidence, and imprecision (where appropriate) [15,16,17].

## 3. Results

**Figure 1:**
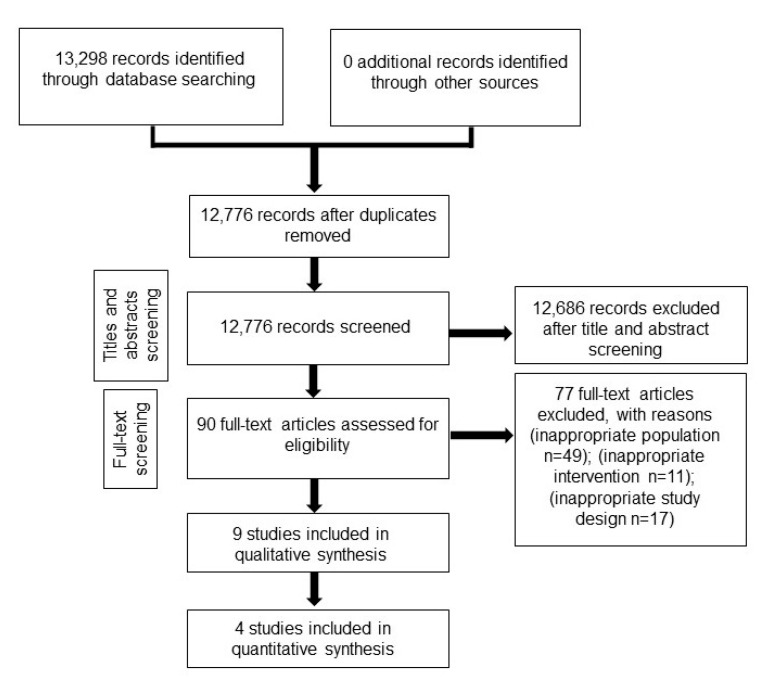
PRISMA flow diagram.

A total of 13,298 records were retrieved from electronic literature searches (see Supplementary Materials FileS1 ). After the removal of duplicates, 12,776 titles and abstracts were screened by two reviewers. Ninety records were found to be potentially eligible and were shortlisted for full-text screening (Figure 1). Thirteen trials met the eligibility criteria [18,19,20,21,22,23,24,25,26,27,28,29,30]. The key data from the included studies are summarized in Table 1. Table 2 presents the details of OMTs. A total of 1393 pediatric patients were included. 

The range of conditions studied included ADHD [18], asthma [23], headache [28], and otitis media [29]. Nine studies were conducted in infants [19,20,21,22,24,25,26,27,30]. Five studies [22,24,25,27,30] were too heterogeneous to be subjected to a meta-analysis and were thus only synthesized narratively. Herzhaft-Le Roy 2017 [22] evaluated the effectiveness of OMT + UC compared with UC alone in 97 infants with biomechanical impairments to suckling. They reported that the intervention (single 30 min session) improved the LATCH score (mean difference (MD) = 1.04; *p* = 0.001) at day 3. The study suffered some methodological weaknesses, including a lack of objective outcome measures or standardized treatment protocol, a small sample, and insufficient power. Danielo Jouhier 2021 [24] evaluated the effectiveness of OMT (two sessions) versus no treatment in 128 infants. They found that the intervention had little or no effect on improving exclusive breast milk feeding at 1 month (odds ratio (OR) = 0.55; 95% confidence intervals (CI) 0.26 to 1.17). This study had no major methodological weaknesses other than not controlling for placebo effects. 

Manzotti 2020 [25] evaluated the effectiveness of a single OMT session (20 min) compared with static touch in 96 preterm infants. They reported that the intervention may improve oxygen saturation (*p* = 0.04) but may have little or no effect on optimizing heart rate (mean change (SD) = 1.2 (13.1)) post-intervention. The study suffered some methodological shortcomings, including a lack of follow-up, a small sample, and significant baseline differences. Raith 2016 [27] evaluated the effectiveness of OMT (20 min/twice a week over three weeks) compared with UC in 30 preterm infants. They reported that the intervention may have little or no effect on improving general movements (*p* > 0.05). The study suffered some methodological weaknesses, including a very small sample, a high drop-out rate, and no control for placebo effects. Accorsi 2014 [18] evaluated the effectiveness of six sessions of 40 min of OMT + UC compared with UC (drug therapy and psychosocial intervention) in 28 children aged 5 to 15 years with attention-deficit/hyperactivity disorder (ADHD). They reported that compared with UC, the intervention reduced the symptoms of ADHD as measured with the Biancardi-Stroppa Modified Bell Cancellation Test. The study suffered some methodological shortcomings, including a very small sample, significant baseline differences, possible confounding effects of UC, and a lack of univariate analyses post-intervention. Jones 2021 [23] evaluated the effectiveness of a single session of 15–20 min + UC versus UC alone in 58 children with asthma. They reported that compared with UC, the intervention had little or no effect on improving lung function. The study suffered some methodological weaknesses, including a small sample, a lack of follow-up, selection bias, and baseline differences in pulmonary function. Rossi 2019 [28] aimed to evaluate the effectiveness of OMT (five sessions over 2 months) versus light touch therapy in 18 teenagers with pediatric headache. This study was ongoing and had no quantitative results. Steele 2014 [29] evaluated the effectiveness of OMT (three weekly visits) versus UC only in 52 young children with otitis media. They reported that compared with UC, the intervention had little or no effect (only within-group differences reported). The study suffered some methodological shortcomings, including a high drop-out rate, a small sample, a lack of power calculation, a high risk of reporting bias, and no control for placebo effects. Castejón-Castejón 2019 [30] evaluated the effectiveness of OMT (1–3 sessions, 30–40 min session once a week for up to 2 weeks versus no treatment) for the treatment of infantile colic. The study found that compared with no treatment, the OMT sessions improved sleeping hours as well as reduced the colic severity and number of crying hours. However, there were some study limitations, such as a small sample, no control for placebo effects, and no blinding of parents, as well as the short duration of the study. 

### 3.1. Meta-Analysis Results: Length of Hospital Stay

**Figure 2:**
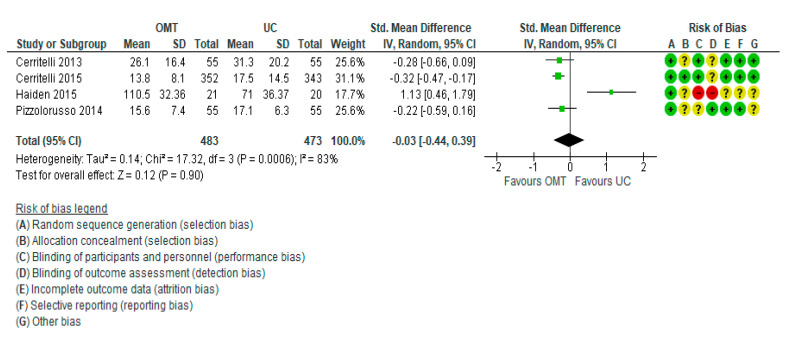
Meta-analysis results. Forest plot for the main comparison [19,20,21,26].

The meta-analysis of four trials [19,20,21,26] showed that when compared with usual care or no intervention, OMT had little or no effect on reducing the length of hospital stay (standardized mean difference (SMD) −0.03; 95% confidence intervals (CI) −0.44 to 0.39)). There was evidence of considerable heterogeneity (Tau^2^ = 0.14; Chi^2^ = 17.32, df = 3 (*p* = 0.0006); I^2^ = 83%; very low certainty evidence) (Table 3, Figure 2). 

### 3.2. Sensitivity Analysis

**Figure 3:**
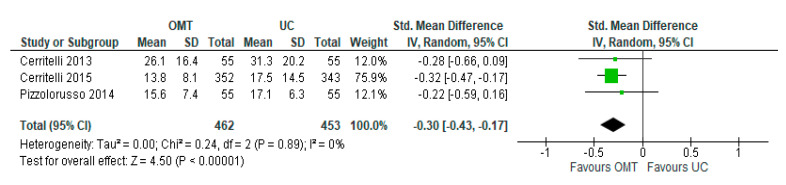
Sensitivity analysis results [19,20,26].

We conducted sensitivity analyses by removing studies with a high risk of bias [21]. The meta-analysis of three trials (judged to have a low or unclear risk of bias) showed that when compared with usual care, OMT marginally reduced the length of hospital stay (SMD −0.30; 95% CI −0.43 to −0.17; low-certainty evidence). There was no evidence of heterogeneity (Tau^2^ = 0.00; Chi^2^ = 0.24, df = 2 (*p* = 0.89); I^2^ = 0%) (Figure 3).

### 3.3. Risk of Bias

**Figure 4:**
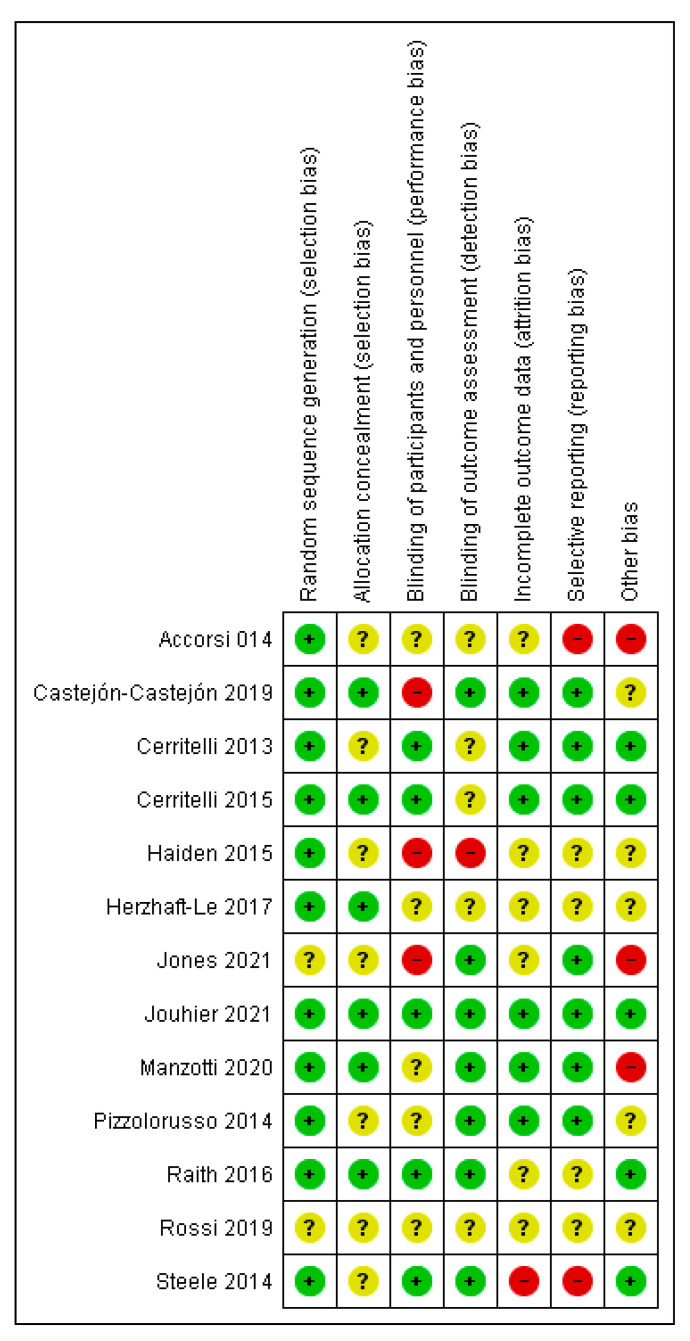
Risk of bias summary: review authors’ judgment of each risk of bias item for each included study [18,19,20,21,22,23,25,26,27,28,29,30].

**Figure 5:**
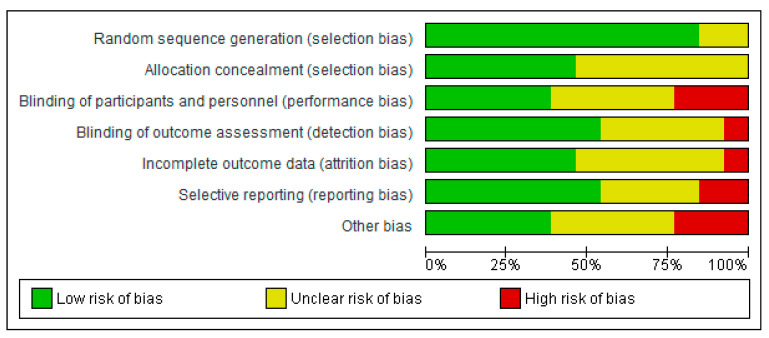
Risk of bias graph. Review authors’ judgment of each risk of bias item presented as percentages of all included studies.

The methodological quality of RCTs published since 2013 seems to have improved. Overall, six studies (46.2%) were judged to have a high risk of bias; six (46.25%) were judged to have an unclear risk of bias, and only one study (7.7%) was judged to have a low risk of bias (Figure 2) [24]. Across all included studies, “other bias”, i.e., baseline differences, were the most common source of bias in 25% of the trials, followed by selective reporting and performance bias. None of the studies were judged to have a high risk of bias for random sequence generation or allocation concealment (Figure 4 and Figure 5). 

#### GRADE Assessments

We downgraded the certainty of evidence for studies’ limitations, inconsistency, and indirectness. The certainty of the evidence was judged to be very low for the following outcome: length of hospital stay. This indicates that we have very little confidence in the effect estimate and that the true effect is likely to be substantially different.

## 4. Discussion

This paper was aimed at updating our previous SR. Thirteen new RCTs were included. Most of them were of poor methodological quality. The meta-analysis of four trials failed to demonstrate that, in preterm infants, OMT reduces the length of hospital stay when compared with usual care. The certainty of the evidence was very low. This is also true for all other outcomes. The only study to be judged to have a low risk of bias failed to demonstrate the effectiveness of OMT in improving breastfeeding at 1 month [24]. For other conditions, such as ADHD, asthma, headache, otitis media, and infantile colic, the findings were contradictory. Adverse effects (AEs) remained poorly reported (or underreported), i.e., four trials failed to mention AEs of OMT [23,25,27,28]. The challenges in designing a methodologically sound RCT were discussed in our original SR [6]. Most of the studies included in our update failed to control for placebo effects, i.e., did not include a separate control group receiving a sham OMT designed to have no real effect. Seven trials (58.3%) compared OMT plus UC to UC; assuming that the extra attention provided through OMT affects the outcome, such a study design would inevitably lead to positive results even in the absence of any specific effects of OMT.

### 4.1. Origin of the Evidence

It is worth noting that 50% of the trials were conducted in Italy by the same group of osteopaths [18,19,20,25,26,28]. Other RCTs originated from Austria [21,27], Canada [22], France [24], and Spain [30], and only two studies came from the US [23,29]. This means that the vast majority of RCTs originated from countries where osteopaths are not conventional but alternative practitioners.

### 4.2. Agreements and Disagreements with Other Reviews 

Our results are in stark contrast with some recently published studies [5,9]. For instance, Lanaro et al. (2017) reported that in preterm infants, OMT reduced the length of hospital stay by 2.71 days (95% CI −3.99, −1.43; *p* < 0.001) [9]. Some of the differences originate from the fact that the authors pooled randomized and longitudinal observational studies [31] together, which inevitably leads to bias [12]. The review by Bagagiolo et al. (2016) was a narrative review, with all of its biases and limitations originating from its non-systematic approach [5]. Similarly, an independent and critical evaluation using the Scottish Intercollegiate Guidelines Network (SIGN) criteria for systematic reviews revealed that a recent SR did not meet the minimum quality criteria of valid research [7]. Finally, a scoping review (limited to US-based observational and experimental studies) by DeMarsh (2021) concluded that little sound evidence exists demonstrating the therapeutic benefit of OMT for pediatric care [8].

### 4.3. Strengths and Limitations 

The strengths of our SR include a comprehensive search strategy as well as our attempt to identify additional and/or unpublished report records. In our update, we adhered to the original search strategy (terms and structure) for retrieving relevant RCTs. The internal validity of this study was ensured by validating data extractions, risk of bias, and GRADE assessments. A multitude of study characteristics was considered for interpreting the results. There were no departures from the pre-planned analyses, and between-study variation was addressed in the synthesis. The limitations of this SR include the considerable amount of heterogeneity of the primary studies and the lack of registration at PROSPERO. We tried to minimize potential biases in the review process by adhering to the guidelines outlined by Higgins 2011 [12]. A major drawback is the paucity of high-quality primary RCTs. This limits the conclusiveness of our findings.

## 5. Conclusions

The quality of the primary trials of OMT has improved during recent years. However, the quality of the totality of the evidence remains low or very low. Therefore, the effectiveness of OMT for selected pediatric populations remains unproven. Further well-designed, robust studies and analyses are required to determine the overall long-term impact of OMT on pediatric patients, taking into consideration confounding and residual co-existing factors. 

## Figures and Tables

**Table 1:** Characteristics of the included studies.

Author Year (Ref.)	n/Characteristics of Participants/ Age or Age Range	Experimental Intervention (Duration/Frequency/Intensity)	Control	Outcome Measure	Main Result (Between-Group Differences)	Effect Estimate	Authors’ Conclusions	AEs/COI	Main Limitations
Accorsi 2014 [18]	28/Children aged 5 to 15 years with attention-deficit/hyperactivity disorder	OMT + UC (6 sessions, 40 min each)	UC only (drug therapy and psychosocial intervention)	Biancardi-Stroppa Modified Bell Cancellation Test: a. accuracy and b. rapidity scores	a. *p* = 0.04 §§ b. *p* = 0.03 §§	1.a. β = 7.948, 95% CI 0.18 to 15.71; 1.b. β = 9.090, 95% CI 0.82 to 17.35	“Participants who received OMT had greater improvement in Biancardi- Stroppa Test scores than participants who received conventional care only”	None reported/not reported	Univariate analyses for post-interventions are missing; very small sample, significant baseline differences; possible confounding effects of UC
Castejón-Castejón 2019 [30]	58/infants aged 0–84 days/infantile colic	OMT (craniosacral therapy) (1–3 sessions, 30–40 min each)	No treatment	1. Crying hours 2. Sleep hours 3. Colic (pain) severity	1. *p* < 0.0005) 2. *p* < 0.0005) 3. *p* < 0.0005)	1. MD = −3.2 (95% CI −3.7, −2.6) at day 24 2. MD = 3.13 (95% CI 2.2, 3.9) at Day 24 3. −18.55 95% CI 21.4, −15.6) at day 24	“Craniosacral therapy appears to be effective and safe for infantile colic by reducing the number of crying hours, the colic severity and increasing the total hours of sleep.”	None reported/not reported	Small sample, no control for placebo effects, no blinding of parents
Cerritelli 2013 [19]	110/preterm infants (34 weeks) *	OMT + UC (20 min)	UC only	1. Length of stay 2. Daily weight gain 3. Costs	1. *p* < 0.03 2. *p* = 0.06§ 3. *p* < 0.001§	MD = −5.20; 95% CI −12.08 to 1.68 (in days) ^	“The present study suggests that OMT may have an important role in the management of preterm infants hospitalization.”	None reported/none declared	Unequal distribution of loss to follow-up; unclear why newborns transferred from another hospital were ineligible
Cerritelli 2015 [20]	695/preterm infants (range: 29 to 37 weeks) *	OMT + UC (30 min/for the entire hospitalization, twice a week)	UC only (20 min)	1. Length of stay 2. Daily weight gain 3. Costs	1. *p* < 0.001 2. n.s. 3. *p* < 0.001	ES = 0.31	“Osteopathic treatment reduced significantly the number of days of hospitalization and is cost-effective on a large cohort of preterm infants”	None reported/none declared	Well-designed and adequately powered, unequal distribution of loss to follow-up, missing details of the OMT
Danielo Jouhier 2021 [24]	128/infants (range 38–42 weeks)	OMT (two sessions)	No OMT	Exclusive breast milk feeding at 1 month	1. n.s.	OR = 0.55; 95% CI 0.26 to 1.17	“OMT did not improve exclusive breast feeding at 1 month.”	None reported/none declared	No control for placebo effects
Haiden 2015 [21]	41/preterm infants (32 weeks) *	Visceral OMT (3 times during their first week of life)	No treatment	1. Time to enteral feedings 2. Length of hospital stay	1. *p* = 0.02 2. n.s.	n.r.	“Infants in the OMT group had a longer time to full enteral feedings and a longer hospital stay what must be interpreted as negative side effect.	None reported/none reported	Small sample, no control for placebo effects, no blinding
Herzhaft-Le Roy 2017 [22]	97/infants with biomechanical impairments to suckling (mean = 15 days)	OMT + UC (4 treatments, once a week for 4 weeks)	UC	LATCH score	*p* = 0.001	MD = 1.04	“Findings support the hypothesis that the addition of osteopathy to regular lactation Consultations is beneficial and safe”	None reported/none declared	Lack of objective outcome measures, treatment protocol not standardized, small sample, underpowered
Jones 2021 [23]	58/children with asthma (mean = 10.8 years)	OMT + UC (single session 15–20 min)	UC	1. FEF 25–75% 2. FVC 3. FEV1 4. FEV-1/FVC ^^	1. *p* = 0.05 2. *p* = 0.26 3. *p* = 0.06 4. *p* = 0.51	1. Mean change + 4.4% 2. Mean change + 2.4% 3. Mean change 2.4% 4. Mean change = 0%	“The benefits of OMT on short term spirometry results in pediatric asthma patients remain unclear”	Not reported/none declared	Small sample, lack of follow-up, long-term benefits/harms unknown, selection bias, baseline differences in pulmonary function
Manzotti 2020 [25]	96/preterm infants (mean (SD) 33.5 (4.3) weeks))	OMT + UC (single session 20 min)	Static touch + UC	1. Heart rate 2. Oxygen saturation	1. n.s. 2. *p* = 0.04	1. Mean change (SD) = 1.2 (13.1) 2. Mean change (SD) = 0.3 (2.4)	“Results from the present study suggest that a single osteopathic intervention may induce beneficial effects on preterm physiological parameters.”	Not reported/none declared	Lack of follow-up; poor biological plausibility, underpowered
Pizzolorusso 2014 [26]	110/preterm infants (range 33.8 and 34.3 weeks) *	OMT (twice per week, 20 min sessions) + UC	UC	Length of stay	*p* < 0.01	Mean = −2.03; 95% CI −3.15 to −0.91	“This study shows evidence that the sooner OMT is provided, the shorter their hospital stay is.”	None reported/none declared	Selection bias; lack of standardized treatment, poor generalizability
Raith 2016 [27]	30/preterm infants (range: 25 and 33 weeks) *	OMT (20 min/twice a week over three weeks)	UC	General movements	*p* > 0.05	n.r.	The primary outcome showed no difference between groups. Craniosacral therapy seems to be safe in preterm infants.	Not reported/none declared	Very small sample, insufficiently powered, high drop-out rate
Rossi 2019 [28]	18/teenagers with pediatric headache	OMT (5 sessions over 2 months)	Light Touch Therapy	Headache frequency, analgesic use, quality of life and adverse events	n.r.	n.r.	“The results are still partial and we need to recruit more patients to have a statistical significance.	Not reported/not declared	Abstract only; no results
Steele 2014 [29]	52/young children with otitis media (range: 6 months to 2 years)	OMT (3 weekly visits)	UC	Change in middle ear effusion over four weeks	n.r. **	OR = 2.98; 95% CI 1.16 to 7.62	“A standardized OMT protocol administered adjunctively with standard care for patients with acute otitis media may result in faster resolution of middle ear effusion […] than UC alone”	None reported/none declared	17.3% drop-out rate; small sample, lack of power calculation, high risk of reporting bias, no control for placebo effects

* = Refers to gestational age; ** = within-group differences reported; ^ = recalculated with RevMan 5.4.; ^^ = all spirometry measures were reported as change scores; § = based on regression analysis; §§ = based on multivariate regression analysis. AE = adverse effect; CI = confidence interval; COI = conflict of interest; ES = effect size; FEF = forced expiratory flow; FEV-1= forced expiration volume in 1st second; FVC = forced vital capacity; MD = mean difference; n.r. = not reported; n.s. = not significant; OMT= osteopathic manipulative treatment; OR = odds ratio; SD = standard deviation; UC = usual care.

**Table 2:** Details of the OMT regimen.

Author Year (Ref)	Details of Treatment (Quote Where Appropriate)
Accorsi 2014 [18]	“Manipulative techniques used included myofascial release, craniosacral, balanced ligamentous tension, and balanced membranous tension”.
Castejón-Castejón 2019 [30]	“The craniosacral treatments were implemented by the main author of the study, a professional craniosacral therapist with 7 years of experience as a paediatric craniosacral therapist and osteopath, and 12 years of experience as a child physiotherapist. The babies received a 30–40 min session once a week (experimental group) or no treatment (control group). Babies in the OMT group received either 1, 2 or 3 CST sessions over a 14-day period.”
Cerritelli 2013 [19]	“The OMT techniques of choice in treating preterm infants are myofascial release, balanced ligamentous/membranous tension, indirect fluidic and v-spread”.
Cerritelli, 2015 [20]	“The treatment included the application of a selected range of manipulative techniques aimed at relieving the somatic dysfunctions. Techniques used were in line with the benchmarks on osteopathic treatment available in the medical literature and were limited to indirect techniques such as: myofascial release and balanced ligamentous/membranous tension.”
Haiden 2015 [21]	“Infants in the intervention group received an osteopathic treatment algorithm within their first 48 h of life according the following protocol adapted from visceral treatment of adults by Barral and Finet”.
Herzhaft-Le Roy 2017 [22]	“[…] after assessing somatic dysfunctions and cranial strains based on tissue texture, tone, asymmetry, and quality of motion, active treatment was carried out, most commonly using techniques such as balanced membranous tension, cranial sutures, and myofascial release.”
Jones 2021 [23]	“Two techniques were used […] Rib raising was performed in the seated position with the physician treating the rib cage bilaterally. […] Suboccipital release was performed for 45 s on a supine patient with the physician’s finger pads contacting the suboccipital musculature”.
Danielo Jouhier 2021 [24]	“The practitioner performed interventions on the part of the body considered appropriate, that is, muscles, bones or viscera […]”.
Manzotti 2020 [25]	“[…] treatment, which is based on the palpatory findings of the initial assessment. It lasted approximately 9 min and aimed at releasing detected changes in the tension and mobility of the tissue. The techniques chosen were those already used in previous studies and demonstrated to be safe in the context of preterm infants.”
Pizzolorusso 2014 [26]	A range of osteopathic techniques were used, including: indirect myofascial release, balanced ligamentous tension or balanced membranous tension.
Raith 2016 [27]	“The 10 step-program was modified as follows: exploration of the cranial system (step 1), treatment of asymmetry (step 2), evaluation of the overlapping of the cranial bones (step 4), exploration of the balance of the membranes of the cranial and spinal dura mater (step 7), exploration and treatment of the sacrum (step 8), and exploration and treatment of the chest (step 9). After the evaluation craniosacral therapy was initiated to achieve the greatest relaxation.”
Rossi 2019 [28]	Abstract only (no details of OMT treatment).
Steele 2014 [29]	“Standardized osteopathic manipulative treatment protocol used in the present study. Adapted from Steele et al. 2010”, which involved 9 techniques.

**Table 3:** Summary of findings.

**Patient or population:** Premature infants						
**Setting:** Neonatology clinics						
**Intervention:** OMT (various techniques)						
**Comparison:** UC						
**Outcomes**	Anticipated absolute effects * (95% CI)		**Relative effect**	**№. of participants**	**Certainty of the evidence**	**Comments**
	**Risk with UC**	**Risk with OMT**	**(95% CI)**	**(studies)**	**(GRADE)**	
**Length of hospital stay**	The mean length of stay was 0	SMD 0.03 lower	-	956		Downgraded for inconsistency, as studies showed contradictory results (I^2^ = 83%). Risk of bias was very high in Haiden 2015. Downgraded for indirectness, as different OMT protocols were used.
		(0.44 lower to 0.39 higher)		(4 RCTs)		
**GRADE Working Group grades of evidence**						
**Very low certainty:** We have very little confidence in the effect estimate: the true effect is likely to be substantially different from the estimate of the effect.						

Footnote: * The risk in the intervention group (and its 95% confidence interval) is based on the assumed risk in the comparison group and the relative effect of the intervention (and its 95% CI). CI = confidence intervals; OMT = osteopathic manipulative treatment; RCT = randomized controlled trial; SMD = standardized mean difference; UC = usual care.

## Supplementary Materials

The following supporting information can be downloaded at: https://www.mdpi.com/article/10.3390/jcm11154455/s1. File S1: PRISMA Checklist, File S2: Search databases. Reference [32] are cited in the supplementary materials.
