# Supplementary material for: Osteopathic Manipulative Treatment for Pediatric Conditions: An Update of Systematic Review and Meta-Analysis
Source: J Clin Med. 2022 Jul 30;11(15):4455. doi: 10.3390/jcm11154455 (PMC9369972; doi:10.3390/jcm11154455)
Supplement: Supplementary file 1 [file jcm-11-04455-s001.zip › File S2.pdf]

Supplement File S2. Search strategy for MEDLINE

#	Query	Results from 9 Nov 2021
1	Osteopath$.ti,ab.	
2	(Osteopath$ adj3 manipulat$).mp.	
3	(Osteopath$ adj5 therap$).mp.	
4	(Osteopath$ adj5 treatment).mp.	
5	(Osteopath$ adj5 medic$).mp.	
6	(Osteopath$ adj5 (viscera$ or cranial or craniosacral or nervous or neural or musculoskelet$ or nonmusculoskeletal or non-musculoskelet$)).ti,ab.	
7	(manual adj2 therap$).ti,ab.	
8	(manual adj2 medic$).ti,ab.	
9	Spencer Technique$.ti,ab.	
10	Jones Technique$.ti,ab.	
11	Strain- Counter Strain.ti,ab.	
12	Positional Release Technique$.ti,ab.	
13	Viscera$ Manipulation$.ti,ab.	
14	Cranial Osteopath$.ti,ab.	
15	Cranio-Sacral Technique$.ti,ab.	
16	Myofascial release.ti,ab.	
17	Soft tissue release.ti,ab.	
18	Muscle energy technique$.ti,ab.	
19	(hand$ adj therap$).ti,ab.	
20	(bone$ adj setter$).ti,ab.	
21	(bone$ adj setter$).ti,ab.	
22	(bodywork adj3 therap$).ti,ab.	
23	(mobili?ation$ adj3 spin$).ti,ab.	
24	(spin $ adj3 adjustment$).ti,ab.	
25	High velocity thrust$.ti,ab.	
26	Low amplitude thrust$.ti,ab.	
27	HVLA.ti,ab.	
28	Manipulat$ therap$.ti,ab.	
29	Manipulat$ joint$.ti,ab.	
30	Subluxation$.ti,ab.	
31	exp osteopathic medicine/	
32	exp manipulation, spinal/	
33	exp musculoskeletal manipulations/	
34	exp manipulation osteopathic/	
35	exp alternative medicine/	
36	exp Complementary Therapies/	
37	manipulation, osteopathic.sh.	
38	osteopathic medicine.sh.	
39	OMT.tw.	

40 osteopath$.tw.

41 1 or 2 or 3 or 4 or 5 or 6 or 7 or 8 or 9 or 10 or 11 or 12 or 13 or 14 or 15 or 16 or 17 or 18 or 19 or 20 or 21 or 22 or 23 or 24 or 25 or 26 or 27 or 28 or 29 or 30 or 31 or 32 or 33 or 34 or 35 or 36 or 37 or 38 or 39 or 40

42 randomized controlled trial.pt.

43 (clin$ adj5 trial$).ti,ab.

44 (((singl$ or doubl$ or tripl$ or trebl$) adj5 blind$ ormask$) or sham).ti,ab.

45 random$.ti,ab.

46 control$.ti,ab.

47 prospectiv$.ti,ab.

48 exp clinical trial/

49 follow-up studies/ or prospective studies/

50 double-blind method/ or random allocation/ or single-blind method/

51 exp Research Design/

52 42 or 43 or 44 or 45 or 46 or 47 or 48 or 49 or 50 or 51

53 exp Infant/

54 (infant$ or infancy or newborn$ or baby$ or babies or neonat $ or preterm$ or prematur$).tw.

55 exp Child/

56 (child$ or schoolchild$ or school age$ or preschool$ or kid or kids or toddler$).tw.

57 Adolescent/

58 (adoles$ or teen$ or boy$ or girl$).tw.

59 Minors/

60 Puberty/

61 (minor$ or pubert$ or pubescen$).tw.

62 exp Pediatrics/

63 (pediatric$ or paediatric$).tw.

64 exp Schools/

65 (nursery school$ or kindergar$ or primary school$ or secondary school$ or elementary school$ or high school$ or highschool$).tw.

66 53 or 54 or 55 or 56 or 57 or 58 or 59 or 60 or 61 or 62 or 63 or 64 or 65 4,863,996

67 41 and 52 and 66

68 limit 67 to yr="2012 - 2021"
